# Isolation and biosynthesis of an unsaturated fatty acid with unusual methylation pattern from a coral-associated bacterium *Microbulbifer* sp.

**DOI:** 10.3762/bjoc.15.225

**Published:** 2019-09-30

**Authors:** Amit Raj Sharma, Enjuro Harunari, Tao Zhou, Agus Trianto, Yasuhiro Igarashi

**Affiliations:** 1 Biotechnology Research Center and Department of Biotechnology, Toyama Prefectural University, 5180 Kurokawa, Imizu, Toyama 939-0398, Japan; 2 Faculty of Fisheries and Marine Sciences, Diponegoro University, Tembalang Campus, St. Prof. Soedarto SH., Semarang 50275, Central Java, Indonesia

**Keywords:** biosynthesis, fatty acid, marine bacteria, methylation, *Microbulbifer*

## Abstract

(2*Z*,4*E*)-3-Methyl-2,4-decadienoic acid (**1**) was identified as a major metabolite from a culture extract of a marine bacterium *Microbulbifer* which was collected from a stony coral *Porites* sp. NMR-based spectroscopic analysis revealed that **1** is an unsaturated fatty acid in which a methyl group is located in an uncommon position as a natural product. Feeding experiments of ^13^C-labeled precursors clarified that ʟ-methionine-derived methylation takes place at the carbon which is derived from the carbonyl carbon of acetate. Compound **1** showed weak growth inhibition against *Saccharomyces cerevisiae*.

## Introduction

Marine microbial symbionts are currently recognized as a reservoir of new bioactive compounds [1]. The most well-studied host animal is the sponge from which a vast array of natural products has been isolated and symbiotic bacteria are suggested to be responsible for the biosynthesis of such natural products [2]. Although it is well established that corals are associated with diverse microbes, coral-associated bacteria and their secondary metabolites have received lesser attention [3]. To date, a couple of new compounds were discovered from soft coral-associated bacteria such as pseudoalteromones from *Pseudoalteromonas* isolated from the cultured octocoral *Lobophytum crassum* [4–5] and macrolactin V from *Bacillus amyloliquefaciens* associated with a gorgonian coral *Junceella juncea* [6], but there is no report on the compounds from stony coral-associated bacteria except our recently published work [7].

**Figure 1:**
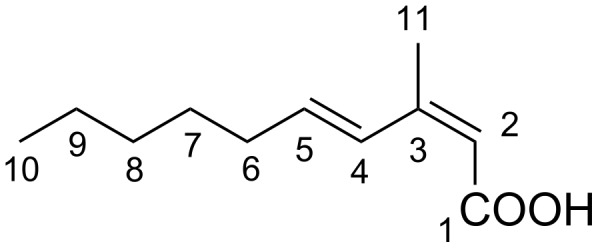
Structure of (2*Z*,4*E*)-3-methyl-2,4-decadienoic acid (**1**).

*Microbulbifer* is a genus of Gram-negative bacteria belonging to the class Gammaproteobacteria [8]. Members of this genus are frequently isolated from halophilic environments including marine solar saltern [9], marine sediment [10], and marine invertebrates [11]. The *Microbulbifer* species are considered to be marine obligate bacteria as they require sodium salt for growth [8]. In our continuing investigation of natural products from marine-derived bacteria, (2*Z*,4*E*)-3-methyl-2,4-decadienoic acid (**1**) was obtained from the culture extract of *Microbulbifer* sp. C4-6 isolated from a stony coral *Porites* sp. (Figure 1). Compound **1** is known as a synthetic compound [12] but this is its first discovery as a natural product. In addition, **1** is biosynthetically unique: it has an uncommon methylation pattern in its carbon chain, derived from the *C*-methylation with ʟ-methionine at a carbon originated from a carbonyl carbon of acetate. In this paper, we report the isolation and structure determination of **1** and its biosynthetic origin proven by the feeding experiments of ^13^C-labeled precursors.

## Results and Discussion

The bacterial strain C4-6 was isolated from a Scleractinian (stony) coral *Porites* sp. On the basis of 16S rRNA gene sequence, this strain was identified as *Microbulbifer*. Strain C4-6 was cultured in A11M seawater medium at 30 °C for five days, and the whole culture broth was extracted with 1-butanol. The extract was consecutively fractionated by normal- and reversed-phase column chromatographies, followed by HPLC purification on a C18 column to yield compound **1**.

**Figure 2:**
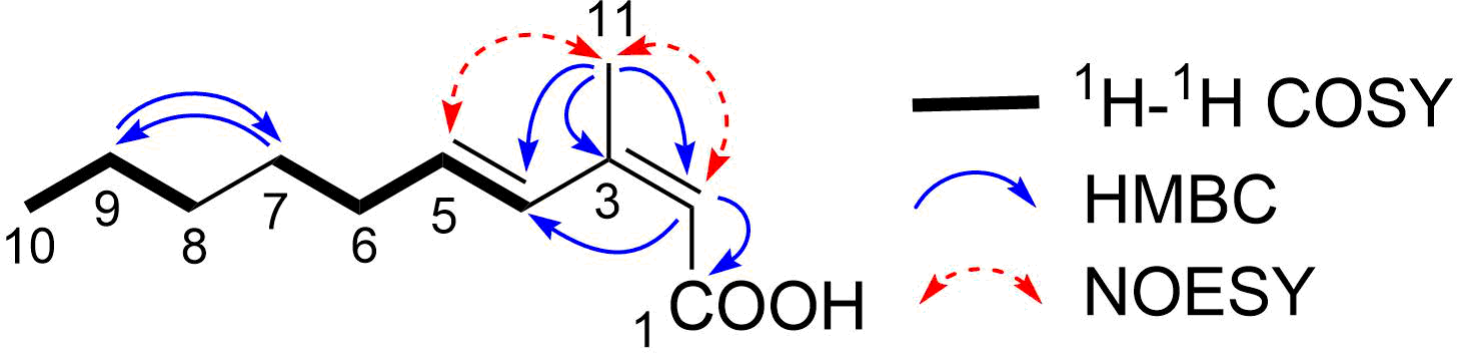
COSY and key HMBC correlations for **1**.

The molecular formula of **1** was determined to be C_11_H_18_O_2_ with three degrees of unsaturation on the basis of its NMR and HR-ESI-TOFMS (*m/z* 181.1230 [M − H]^−^; calcd for C_11_H_17_O_2_, 181.1229) data. The UV spectrum of **1** in methanol exhibited an absorption maximum at 262 nm. The IR absorption bands at 1678 and 2800–3200 cm^−1^ suggested the presence of carboxyl group. The ^1^H and ^13^C NMR data of **1** (Table 1) showed the presence of 11 carbon signals, which were identified by the assistance of a DEPT135 spectrum as two methyls, four sp^3^ methylenes, three sp^2^ methines (δ_C_ 140.6, 127.7, 115.1), and two sp^2^ nonprotonated carbons including one carbonyl group (δ_C_ 171.3, 153.9). Besides, the ^1^H NMR spectrum showed characteristic resonances, especially for two methyl groups at δ_H_ 2.02 (3H, s) and 0.90 (3H, t) in the highfield region and for multiple methylene signals, suggesting the presence of an alkyl chain. COSY analysis established two separate spin systems, H4/H5/H6/H7 and H8/H9/H10. These partial structures were joined by the mutual HMBC correlations between H7 and H9. Furthermore, long-range correlations from H11 to C2, C3, and C4 and from H2 to C1, C3, and C4 established the carbon connectivity among these carbons to complete the structure of **1** (Figure 2). The double bond geometry was determined on the basis of NOESY analysis and ^3^*J*_HH_ coupling constants. NOE was detected between H2 and H11 but not between H2 and H4, reflecting the *Z-*configuration for the C2–C3 double bond. Similarly, NOESY correlations for H4/H6 and H5/H11 reflected the *E*-configuration for the C4–C5 double bond, which was corroborated by ^3^*J*_HH_ vicinal coupling constant (*J*_H4,H5_ = 15.8 Hz).

**Table 1:** ^1^H and ^13^C NMR data for compound **1** in CDCl_3_.

position	δ_C_^a^	δ_H_ mult (*J* in Hz)^b^	HMBC^b,c^

1	171.3, C		
2	114.8, CH	5.63, s	1, 3, 4, 11
3	153.8, C		
4	127.5, CH	7.55, d (15.8)	2, 3, 6, 11
5	140.3, CH	6.22, dt (15.8, 7.2)	3, 6, 7
6	33.3, CH_2_	2.24, dt (7.2, 7.2)	4, 5, 7, 8
7	28.7, CH_2_	1.46, m	5, 8, 9
8	31.4, CH_2_	1.32^d^, m	6, 7, 10
9	22.4, CH_2_	1.32^d^, m	
10	13.9, CH_3_	0.90, t (7.1)	8, 9
11	21.3, CH_3_	2.02, s	2, 3, 4

^a^Recorded at 125 MHz (reference δ_C_ 77.0). ^b^Recorded at 500 MHz (reference δ_H_ 7.26). ^c^HMBC correlations are from proton(s) stated to the indicated carbon. ^d^Overlapping signals.

**Figure 3:**
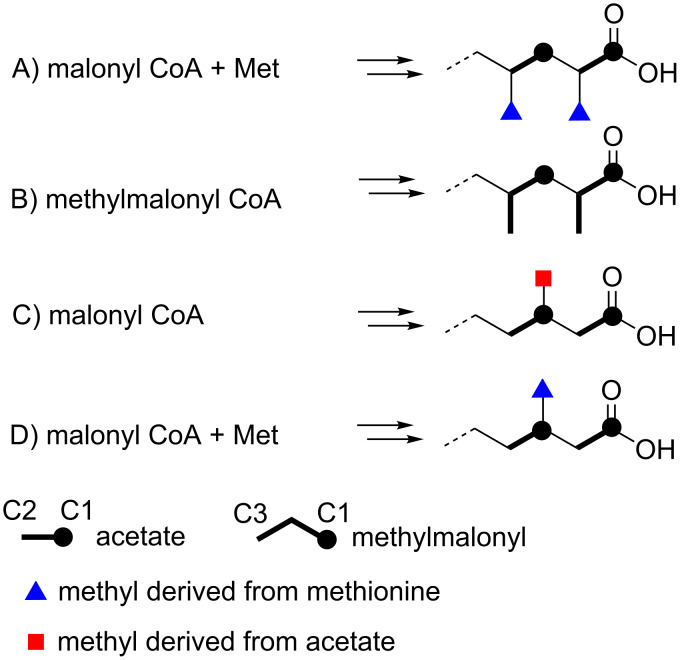
Methylation pattern that can occur in fatty acids and polyketides.

**Scheme 1:**
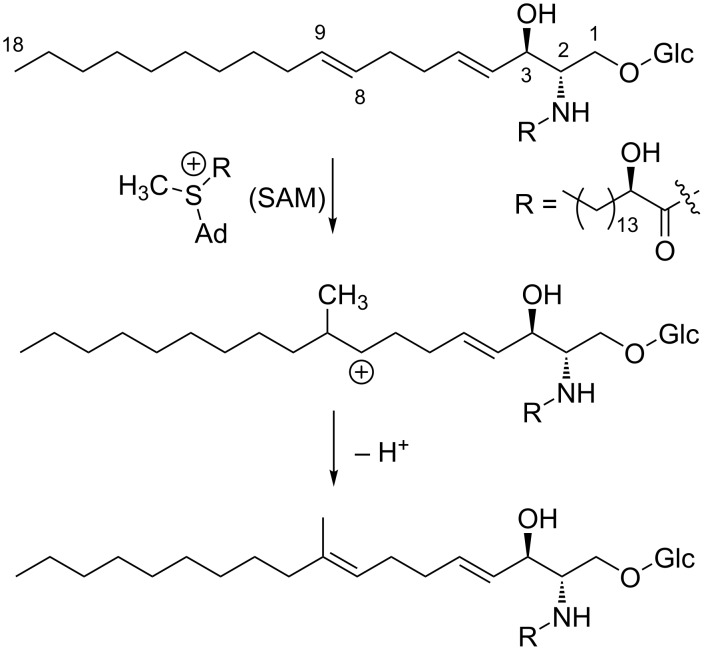
*C*-Methylation of alkenyl carbon in sphingolipid biosynthesis.

In fungi and certain kinds of bacteria, methyl substituents in the fatty acid carbon chain or the polyketide chain are derived from the methyl group of *S*-adenosylmethionine (SAM) (Figure 3A) [13–14]. This methylation reaction usually occurs at the nucleophilic carbons originated from the methyl carbon of acetate (C2) since SAM acts as an electrophilic methyl donor. In most of the bacteria including actinomycetes, methyl branching in polyketide chain is derived from methylmalonyl CoA, thereby the methylation position must be also the α-position (C2) of the acetate unit (Figure 3B) [13]. In contrast, methylation at the carbons derived from the carbonyl carbon of the acetate (C1) is quite unusual in fatty acids and polyketides. This unusual methylation pattern is reported for the polyketides of eukaryotic algae dinoflagellates. Some of the methyl groups in dinoflagellate compounds are positioned at the carbons derived from the carbonyl carbon of acetate (C1). Precursor labeling studies indicated that the origin of the methyl carbon is not SAM but one carbon fragment derived from the cleavage of another acetate unit (Figure 3C) [15]. The only single example of *C*-methylation with SAM at a carbon derived from the carbonyl carbon of acetate (C1; Figure 3D) is reported for sphingolipid biosynthesis in the yeast, *Pichia pastoris* [16]. SAM-dependent *C*-methylation takes place at the alkenyl carbon C9 of glucosylceramide, yielding a cationic intermediate, from which deprotonation occurs at C9 to give an internal olefin (Scheme 1).

**Figure 4:**
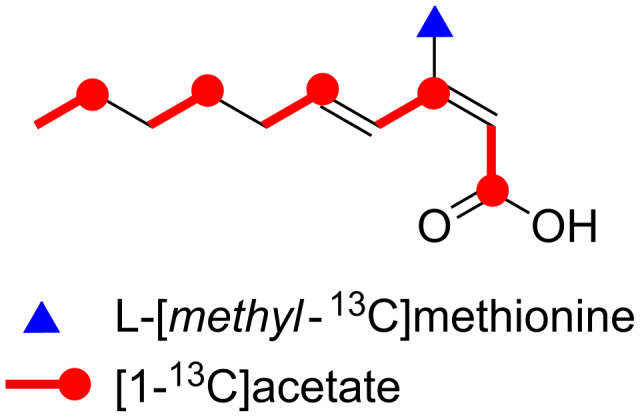
Incorporation of ^13^C-labeled precursors into **1**.

**Scheme 2:**
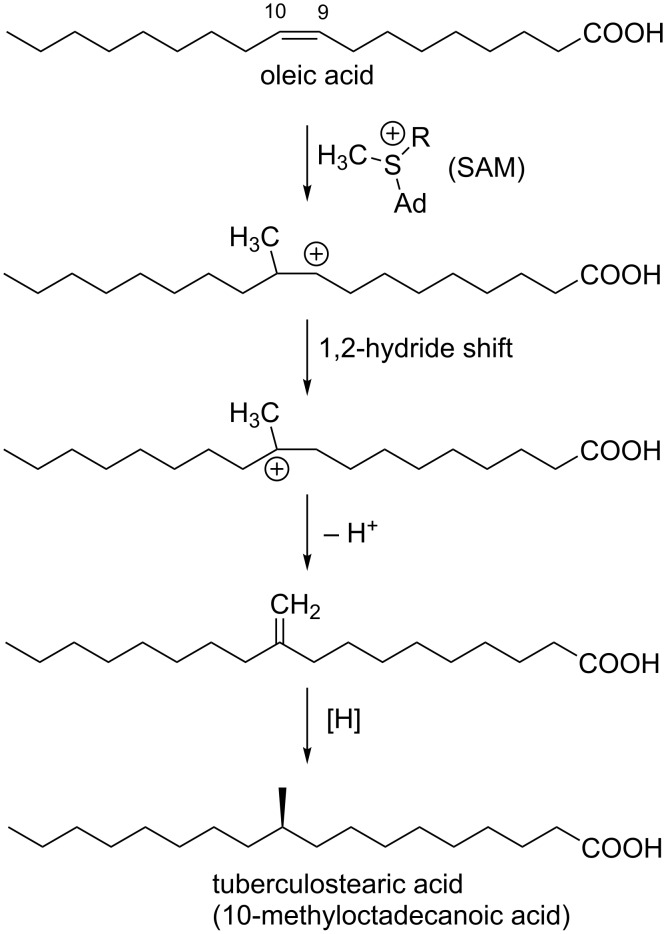
Biosynthesis of tuberculostearic acid in *Mycobacterium*.

**Scheme 3:**
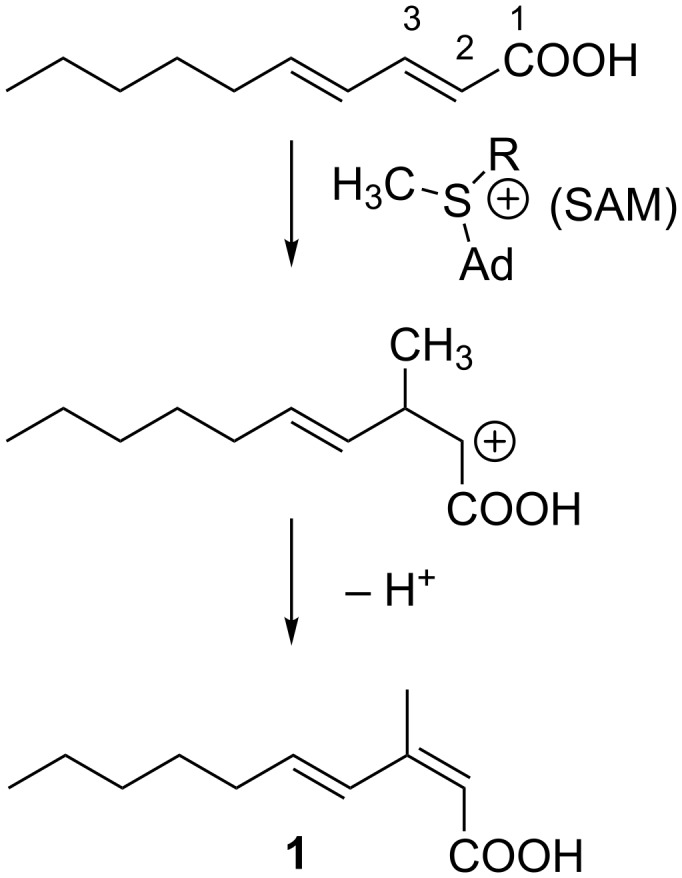
Possible methylation mechanism for compound **1**.

In the case of **1**, methylation at the C3 carbon is inconsistent with the regular methylation pattern that occurs in fatty acids synthesized by the FAS (fatty acid synthase) or polyketides from the PKS (polyketide synthase) system of bacterial groups. Therefore, the biosynthetic origin of **1** was investigated by feeding experiments of ^13^C-labeled precursors (Table 2). Firstly, in order to verify the origin of the carbon backbone, [1-^13^C]acetate was fed to the culture which gave a high enrichment of the carbons at C1, C3, C5, C7, and C9 (Figure 4). A feeding experiment with ʟ-[*methyl*-^13^C]methionine was then carried out. The high level of enrichment was observed only for C11 in the ^13^C NMR spectrum, thereby confirming that the methyl group is derived from methionine via SAM. In addition to the fungal sphingolipids described above, methyl branches with SAM-origin are also found in some bacterial fatty acids like tuberculostearic acid, a major constituent of mycobacterial membrane phospholipids [17]. Methylation at the olefinic double bond of oleic acid part in phospholipids is catalyzed by SAM-dependent methyltransferase, followed by 1,2-hydride shift and deprotonation, and a subsequent reduction of the *exo*-methylene intermediate gives rise to a methyl group (Scheme 2) [18]. The presence of the *exo*-methylene intermediate was experimentally proved but the enzyme responsible for the double bond reduction has not been identified [18]. Based on the *C*-methylation pattern found in the biosynthesis of sphingolipid and tuberculostearic acid, we propose a possible mechanism of methylation for compound **1** although further chemical/biochemical investigation is required (Scheme 3). First, 2,4-decadienoic acid is formed and methylation takes place at C3 to give a putative cationic intermediate. The following proton loss from C3 is probable to give directly **1** in a similar fashion to the glucosylceramide biosynthesis. Branched chain fatty acids are commonly present in cell membranes, and mono-methylation at the *iso* or *anteiso* position is the most common [19]. Branching at other positions is less common but represents a significant proportion of branched-chain fatty acids in some organisms [19]. Tuberculostearic acid (10-methyloctadecanoic acid (18:1 me(10)), 10- methylhexadecanoic acid (16:1 me(10)), and 10-methylnonadecanoic acid (19:1 me(10)) are the major constituents of the cell wall of *Mycobacterium phlei* [20]. C9-Methylated glucosylceramides also possess a methyl-branching in the middle of the aliphatic carbon chain of the sphingosine part. C9-Methylated sphingolipids have not been found from plants and animals but are widely produced by many fungi and are thought to play an important role in the interaction between fungi and their host organisms. C9-Methylated sphingolipids are also found from some marine invertebrates such as sea anemone and starfish although their biological function is still unclear in these organisms. *C*-Methylation at C9 of fungal sphingosines is similarly catalyzed by SAM-dependent methyltransferases [16]. The methylation takes place at the alkenyl carbon (C9) which is originally derived from the carbonyl carbon of an acetate unit: C1 and C2 of sphingosines are derived from ʟ-serine and carbons from C3 to C18 are from palmitoyl-CoA. To the best of our knowledge, compound **1** is the first example of a simple fatty acid in which the carbonyl carbon of an acetate unit is methylated with SAM (Figure 3D).

**Table 2:** Incorporation of ^13^C-labeled precursors into **1**.

		relative enrichment^a^	
position	δ_C_^b^	[1-^13^C]acetate	ʟ-[*methyl*-^13^C]-methionine

1	171.3	**8.99**	1.10
2	114.8	0.91	1.12
3	153.8	**10.13**	0.84
4	127.5	1.00	1.00
5	140.3	**11.19**	0.99
6	33.3	1.15	1.12
7	28.7	**11.35**	1.12
8	31.4	1.19	1.20
9	22.4	**12.01**	1.13
10	13.9	1.37	1.17
11	21.3	1.20	**17.74**

^a^The ^13^C signal intensity of each peak in the labeled **1** divided by that of the corresponding signal in the unlabeled, normalized to the peak area of C4 to give an enrichment ratio for enriched peak. The numbers in bold type indicate ^13^C-enriched atoms from ^13^C-labeled precursors. ^b^Recorded at 125 MHz (reference δ_C_ 77.0).

Compound **1** was not active against *Staphylococcus aureus* FDA209P JC-1*, Micrococcus luteus* ATCC9341, *Bacillus subtilis* ATCC6633, *Escherichia coli* NIHJ JC-2, *Ralstonia solanacearum* SUPP1541, *Rhizobium radiobacter* NBRC14554, and *Candida albicans* NBRC0197 (MIC > 100 μg/mL) but weakly active against *Saccharomyces cerevisiae* S100 (MIC 100 μg/mL).

## Conclusion

In summary, chemical investigation of metabolites in a marine-derived *Microbulbifer* sp. led to the discovery of a unique unsaturated fatty acid, (2*Z*,4*E*)-3-methyl-2,4-decadienoic acid (**1**) in which the carbon originated from the carbonyl carbon of an acetate unit is methylated, providing a quite rare case of *C*-methylation pattern in fatty acid/polyketide biosynthesis. Compound **1** and its (2*E*,4*E*)-isomer were reported previously as synthetic compounds but this is the first finding of **1** as a natural product [12,21]. Very recently, benzoate derivatives were reported from a marine-derived *Microbulbifer* [22]. Therefore, this is the second report on the small molecule from this underexplored taxon. According to the genome sequence database, biosynthetic genes for NRPS and siderophore are present in *Microbulbifer* species which will be pursued in our future investigation.

## Experimental

### General experimental procedures

The UV spectrum was recorded on a Shimadzu UV-1800 spectrophotometer. The IR spectrum was measured on a Perkin-Elmer Spectrum 100. NMR spectra were obtained on a Bruker AVANCE 500 spectrometer in CDCl_3_ using the signals of the residual solvent proton (δ_H_ 7.26) and carbon (δ_H_ 77.0) as internal standards. HR-ESI-TOFMS were recorded on a Bruker microTOF focus. Sodium [1-^13^C]acetate was purchased from Cambridge Isotope Laboratories, Inc., and ʟ-[*methyl*-^13^C]methionine from Sigma-Aldrich Co. LLC.

### Microorganism

Strain C4-6 was isolated from a cultured stony coral (*Porites* sp.) obtained from an aquarium vendor in Nagasaki, Japan. The coral specimen was washed with 70% ethanol and then washed with sterile natural seawater. A fragment of the coral (ca. 1 g) was homogenized by mortar and pestle with an equal amount of sterile natural seawater (1 mL), and 10-fold serial dilution was carried out up to 10^−5^ and 0.1 mL of each dilution was spread onto marine agar 2216 (Difco). The plates were kept at 23 °C, and a single colony was repeatedly transferred onto the same agar medium to obtain the pure isolate of strain C4-6. The strain was identified as a member of the genus *Microbulbifer* on the basis of 99.0% similarity in the 16S rRNA gene sequence (1405 nucleotides; DDBJ accession number LC456787) to *Microbulbifer variabilis* Ni-2088^T^ (accession number AB167354).

### Fermentation

The producing strain C4-6 was maintained on marine agar 2216 (Difco). A single colony of strain C4-6 was inoculated into a 500 mL K-1 flask containing 100 mL of marine broth 2216 (Difco) as a seed culture. The seed culture was incubated at 30 °C on a rotary shaker at 200 rpm for 2 days. Three mL of seed culture was inoculated into twenty-five 500 mL K-1 flasks each containing 100 mL of A11M production medium, which consists of glucose 0.2%, soluble starch 2.5%, yeast extract 0.5%, polypeptone (Wako Pure Chemical Industries, Ltd.) 0.5%, NZ-amine (Wako Pure Chemical Industries, Ltd.) 0.5%, CaCO_3_ 0.3%, and Diaion HP-20 (Mitsubishi Chemical Co.) 1% in natural seawater (collected from Toyama Bay, Japan). The pH of the medium was adjusted to 7.0 before sterilization. The inoculated flasks were incubated at 30 °C on a rotary shaker at 200 rpm for 5 days.

### Extraction and isolation

After fermentation, 100 mL of 1-butanol was added to each production culture flask, and the flasks were allowed to shake for 1 h on a rotary shaker at 200 rpm. The mixture was centrifuged at 6000 rpm for 10 min in order to separate organic layer and aqueous layer. The organic layer was concentrated in vacuo to afford 3.2 g of extract from 2.5 L of production culture. The extract was fractionated by silica gel column chromatography with a step gradient of CHCl_3_/MeOH (1:0, 20:1, 10:1, 4:1, 2:1, 1:1, and 0:1 v/v). Fraction 3 (10:1) was concentrated to give a brown oil (646 mg), which was then fractionated by reversed-phase ODS column chromatography with a gradient of MeCN/0.1% HCO_2_H (2:8, 3:7, 4:6, 5:5, 6:4, 7:3, and 8:2 v/v). Fraction 6 (7:3) was concentrated and extracted with EtOAc. The organic layer was dried over anhydrous Na_2_SO_4_, filtered, and concentrated to give a semi-pure material (21 mg). Final purification was achieved by preparative HPLC (Cosmosil Cholester 5 µm, 10 × 250 mm, 4 mL/min, UV detection at 254 nm) with an isocratic elution of MeCN/0.1% HCO_2_H solution (50:50) to yield (2*Z*,4*E*)-3-methyl-2,4-decadienoic acid (**1**, 8.0 mg, *t*_R_ 20.5 min).

(2*Z*,4*E*)-3-Methyl-2,4-decadienoic acid (**1**): colorless amorphous solid; UV (MeOH) λ_max_ (log ε) 262 (4.16) nm; IR (ATR) *ν*_max_ 2952, 2923, 2852, 2585, 1678, 1662 cm^−1^; ^1^H and ^13^C NMR data, see Table 1; HR-ESI-TOFMS *m/z* 181.1230 [M − H]^−^ (calcd for C_11_H_17_O_2,_ 181.1229).

### Feeding experiment

Feeding experiments were carried out using ^13^C-labeled precursors, sodium [1-^13^C]acetate and ʟ-[*methyl*-^13^C]methionine. The fermentation, extraction and purification of labeled compounds were performed in the same manner as describe for the unlabeled compound. After 24 h of the inoculation into the production medium from the seed culture, supplementation of ^13^C-labeled precursors was commenced and carried out for four times at 24 h interval. After further 24 h incubation, the whole culture broth was extracted with 1-butanol.

1) Sodium [1-^13^C]acetate: ^13^C-Labeled **1** (1.6 mg) was obtained from 1 L of culture supplemented with sodium [1-^13^C]acetate (total 800 mg; 20 mg × 10 flasks × 4 days). The ^13^C NMR spectrum showed enriched signals at δ 170.2, 153.8, 140.3, 28.7, and 22.4 ppm.

2) ʟ-[*Methyl*-^13^C]methionine: ^13^C-Labeled **1** (0.9 mg) was obtained from 1 L of culture supplemented with ʟ-[*methyl*-^13^C]methionine (total 80 mg; 2.0 mg × 10 flasks × 4 days). The ^13^C NMR spectrum showed an enriched signal at δ 21.3 ppm

### Antimicrobial assay

Antimicrobial activity was evaluated by the liquid microculture method using round-bottomed 96-well microtiter plates against six bacteria, *Bacillus subtilis* ATCC6633, *Micrococcus luteus* ATCC9341, *Staphylococcus aureus* FDA209P JC-1, *Ralstonia solanacearum* SUPP1541, *Rhizobium radiobacter* NBRC14554, *Escherichia coli* NIHJ JC-2, and two yeasts *Candida albicans* NBRC0197 and *Saccharomyces cerevisiae* S100 as indication strains. Tryptic soy broth (Difco) and Sabouraud dextrose broth (Difco) were used for bacteria and yeasts, respectively. Compound **1** and reference drugs, kanamycin sulfate for bacteria and amphotericin B for yeasts, were made in 2-fold dilution series along the longer side of the plates by sequential transfer of 100 μL aliquots between the adjacent wells, to which the same amount of medium was pre-dispensed. To each well was added a 100 μL suspension of the indication strains prepared at 0.5 McFarland (≈10^8^ cfu/mL) from a culture at the logarithmic growth phase. The solvent vehicle added to the top rows was set at the 0.5% of the final culture volume to avoid the effect on the growth of microbes. The plates were incubated for 48 h at 37 °C for bacteria and at 32 °C for yeasts. The tests were done in triplicate and the MIC values were read from the lowest drug concentrations at which no growth was observed.

## Supporting Information

File 1 1D and 2D NMR spectra of **1**; ^13^C NMR spectra of ^13^C-labeled **1**.
