# Supplementary material for: Isolation and biosynthesis of an unsaturated fatty acid with unusual methylation pattern from a coral-associated bacterium Microbulbifer sp
Source: Beilstein J Org Chem. 2019 Sep 30;15:2327–32. doi: 10.3762/bjoc.15.225 (PMC6808205; doi:10.3762/bjoc.15.225)
Supplement: File 1 — 1D and 2D NMR spectra of 1; 13C NMR spectra of 13C-labeled 1. [file Beilstein_J_Org_Chem-15-2327-s001.pdf]

BEILSTEIN JOURNAL OF ORGANIC CHEMISTRY

Supporting Information

for

Isolation and biosynthesis of an unsaturated fatty acid with unusual methylation pattern from a coral-associated bacterium *Microbulbifer* sp.

Amit Raj Sharma, Enjuro Harunari, Tao Zhou, Agus Trianto and Yasuhiro Igarashi

Beilstein J. Org. Chem. **2019,** *15,* 2327–2332. doi:10.3762/bjoc.15.225

1D and 2D NMR spectra of 1; ^{13}C NMR spectra of ^{13}C-labeled 1

License and Terms: This is a supporting information file under the terms of the Creative Commons Attribution License (http://creativecommons.org/licenses/by/4.0). Please note that the reuse, redistribution and reproduction in particular requires that the authors and source are credited.
The license is subject to the *Beilstein Journal of Organic Chemistry* terms and conditions: (https://www.beilstein-journals.org/bjoc)

Table of contents

Figure S1. UV spectrum of (2*Z*,4*E*)-3-methyl-2,4-decadienoic acid (**1**)
Figure S2. High resolution ESI-TOF mass spectrum of **1**
Figure S3. IR spectrum of **1**
Figure S4. ^{1}H NMR spectrum of **1** (500 MHz, $CDCl_3$)
Figure S5. ^{13}C NMR spectrum of **1** (125 MHz, $CDCl_3$)
Figure S6. DEPT135 spectrum of **1** (125 MHz, $CDCl_3$)
Figure S7. COSY spectrum of **1** (500 MHz, $CDCl_3$)
Figure S8. HSQC spectrum of **1** (500 MHz, $CDCl_3$)
Figure S9. HMBC spectrum of **1** (500 MHz, $CDCl_3$)
Figure S10. NOESY spectrum of **1** (500 MHz, $CDCl_3$)
Figure S11. ^{13}C NMR spectra of **1** labeled with [1-^{13}C]acetate and L-[*methyl*-^{13}C]methionine (125 MHz, $CDCl_3$)

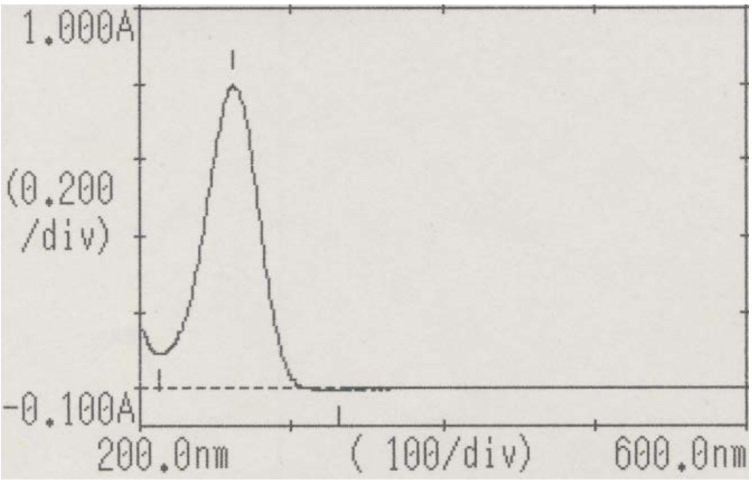

Figure S1. UV spectrum of (2*Z*,4*E*)-3-methyl-2,4-decadienoic acid (**1**)

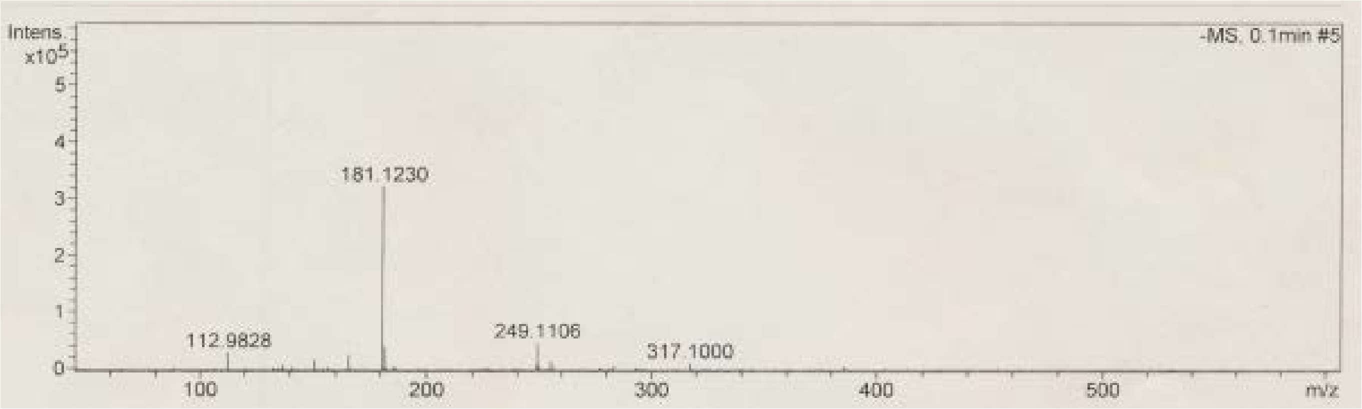

Figure S2. High resolution ESI-TOF mass spectrum of **1**

(ATR)

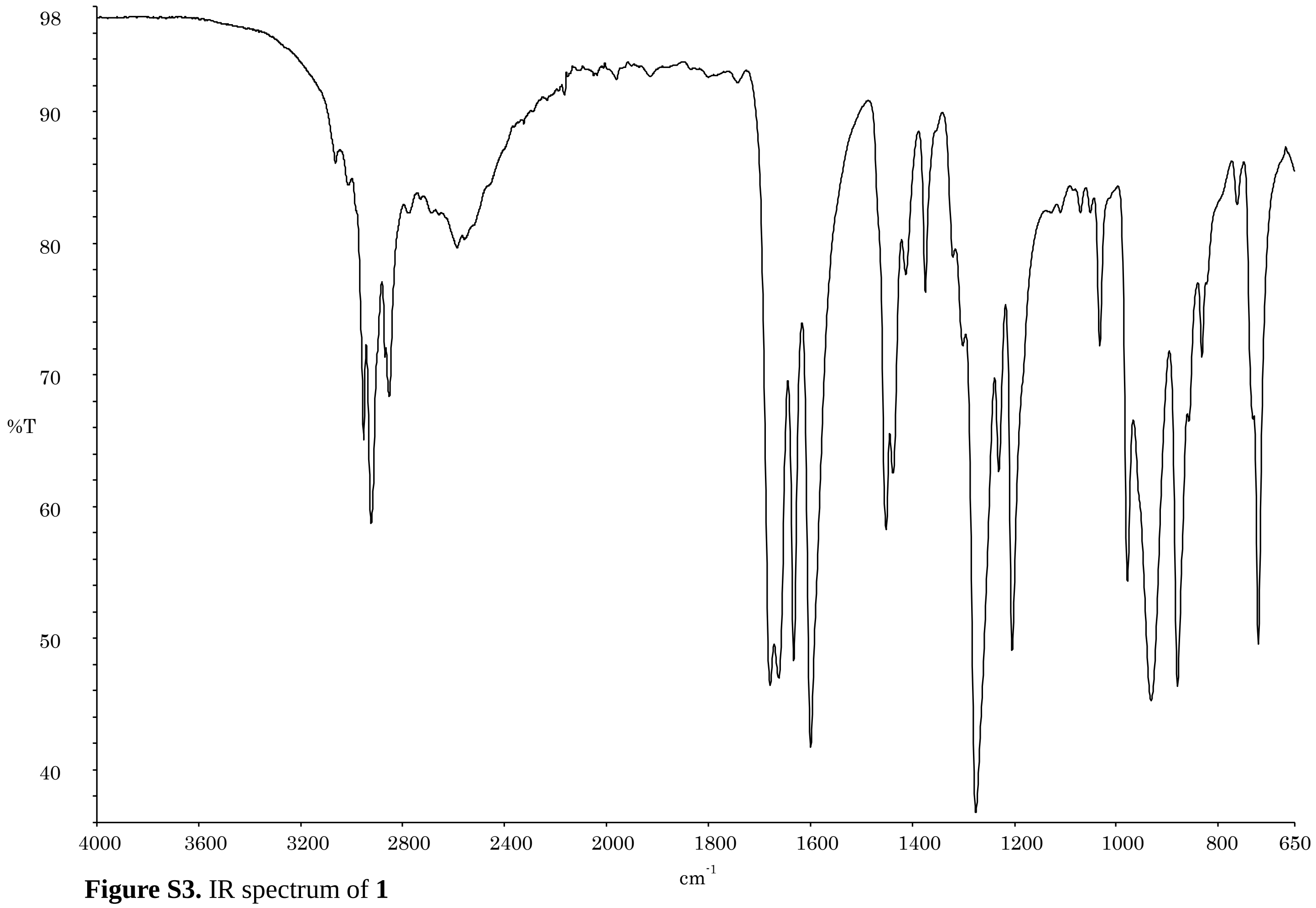

Figure S3. IR spectrum of **1**

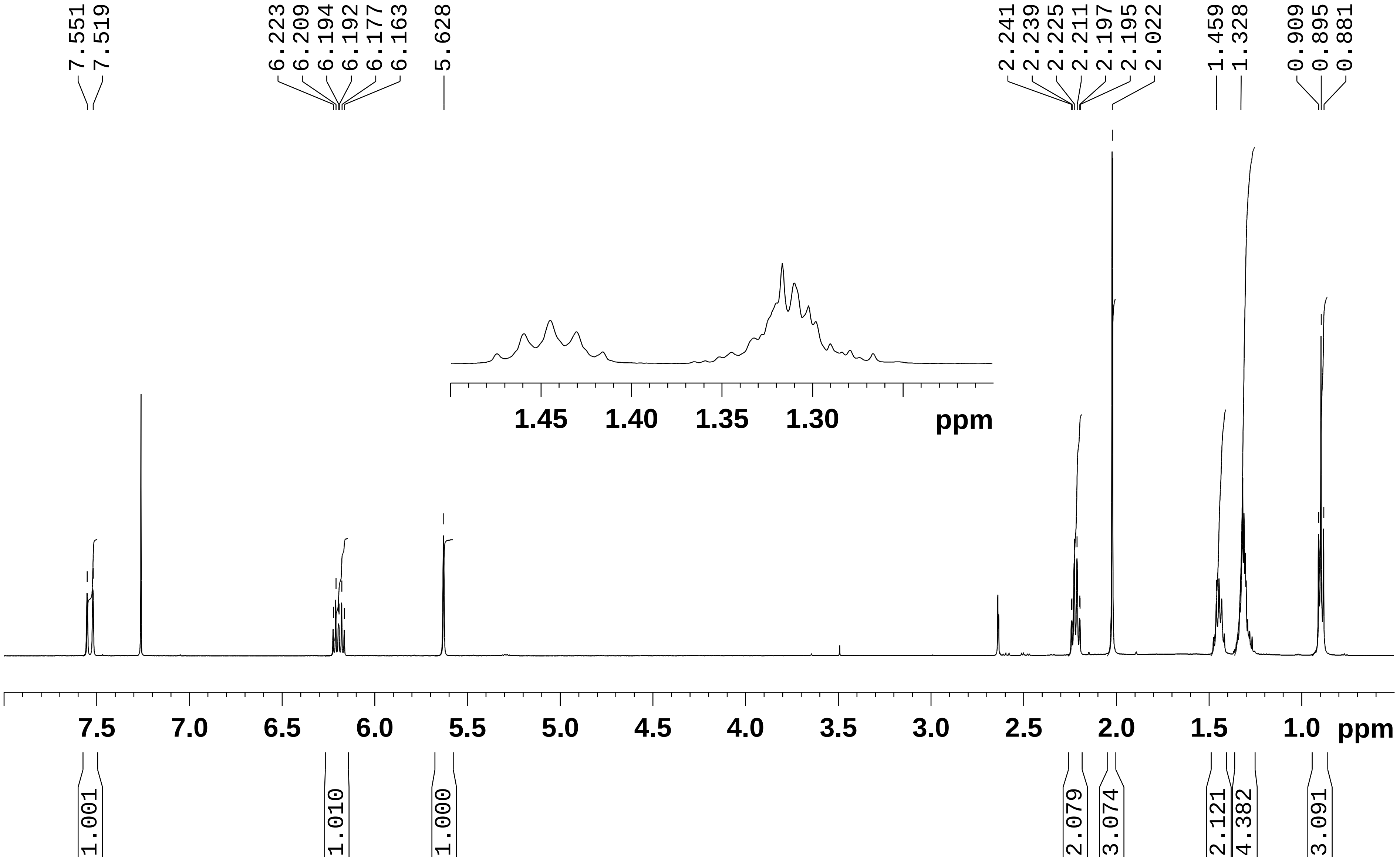

Figure S4. ^{1}H NMR spectrum of **1** (500 MHz, $CDCl_3$)

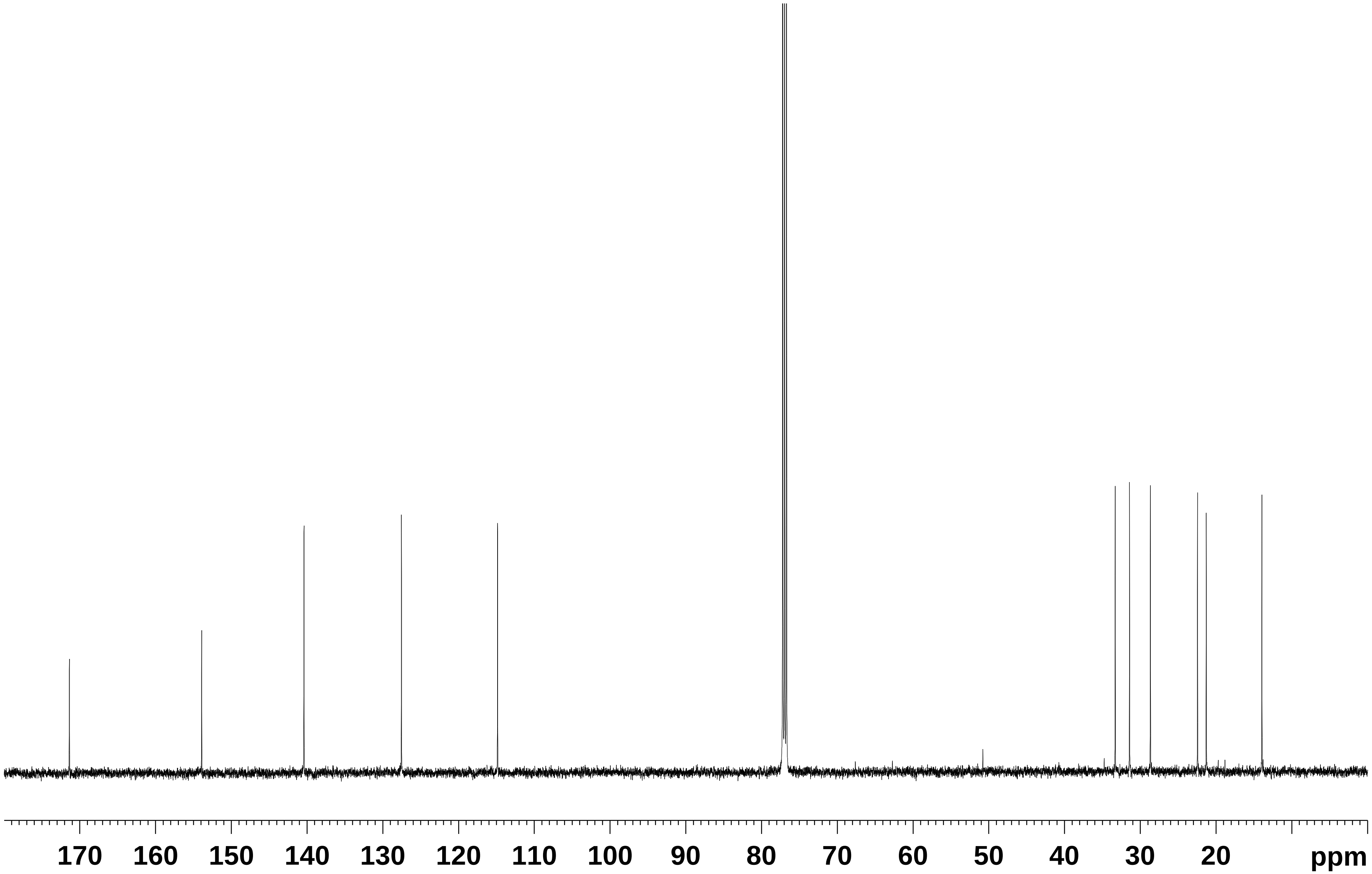

Figure S5. ^{13}C NMR spectrum of **1** (125 MHz, $CDCl_3$)

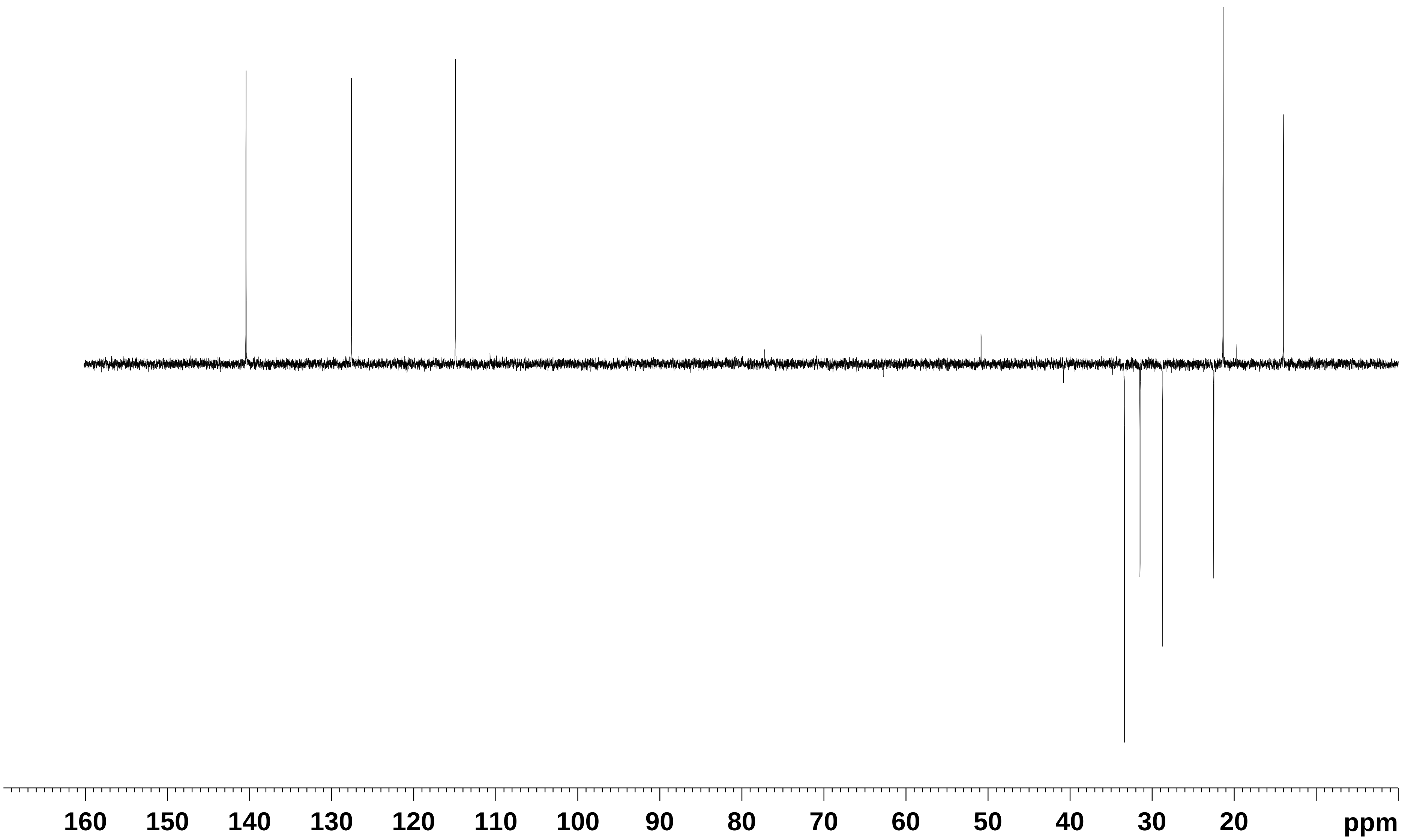

Figure S6. DEPT135 spectrum of **1** (125 MHz, $CDCl_3$)

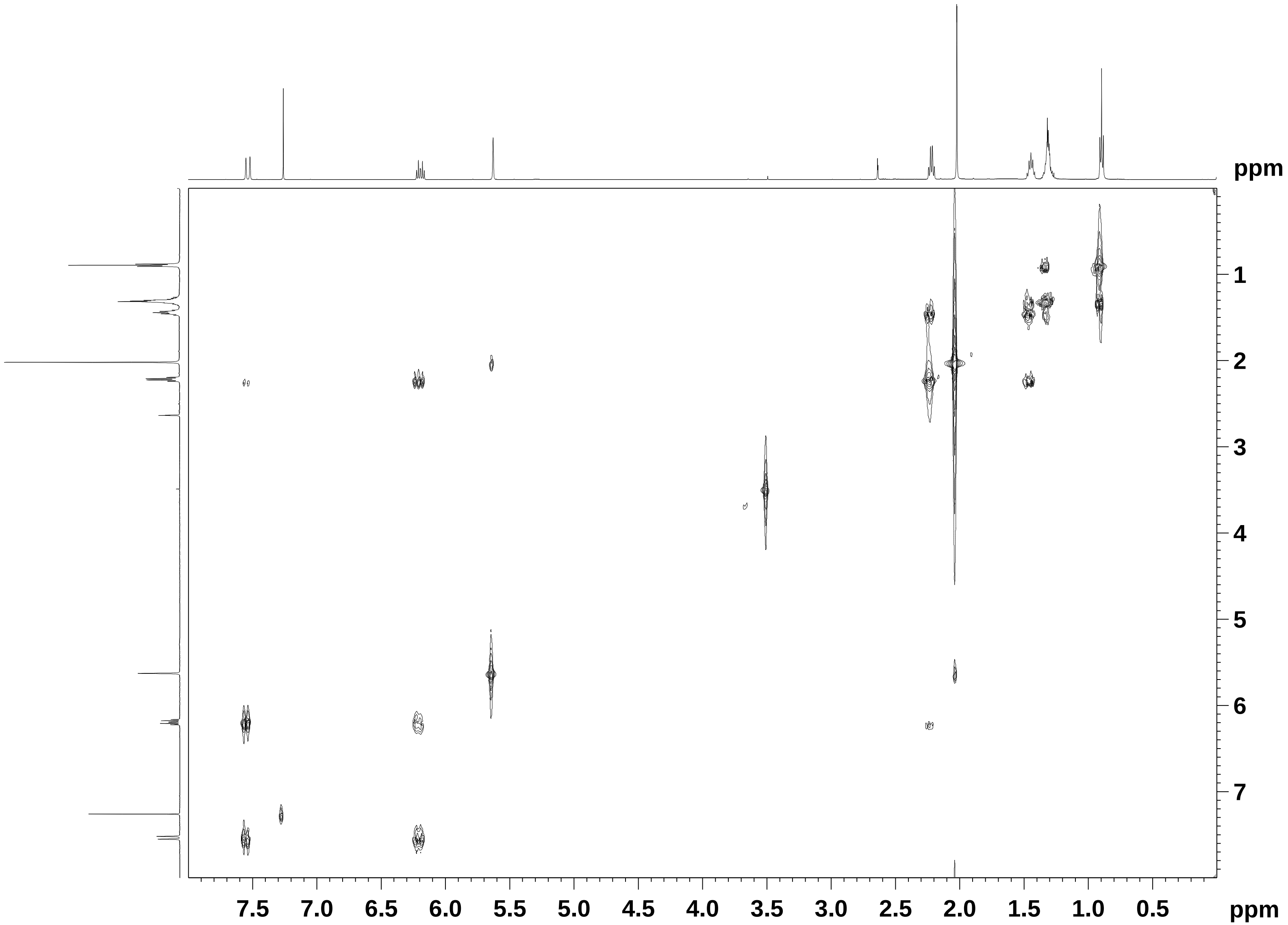

Figure S7. COSY spectrum of **1** (500 MHz, $CDCl_3$)

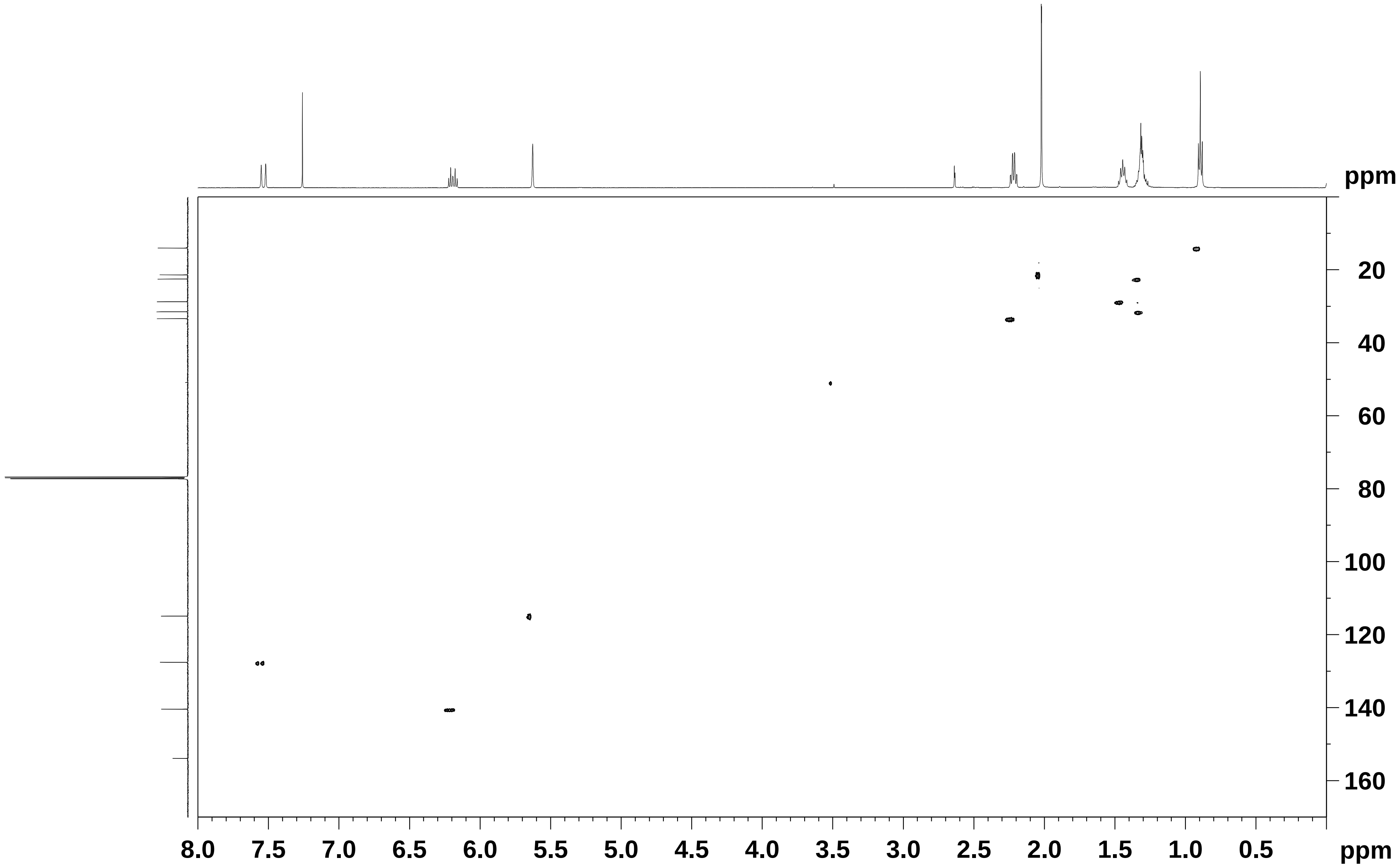

Figure S8. HSQC spectrum of **1** (500 MHz, $CDCl_3$)

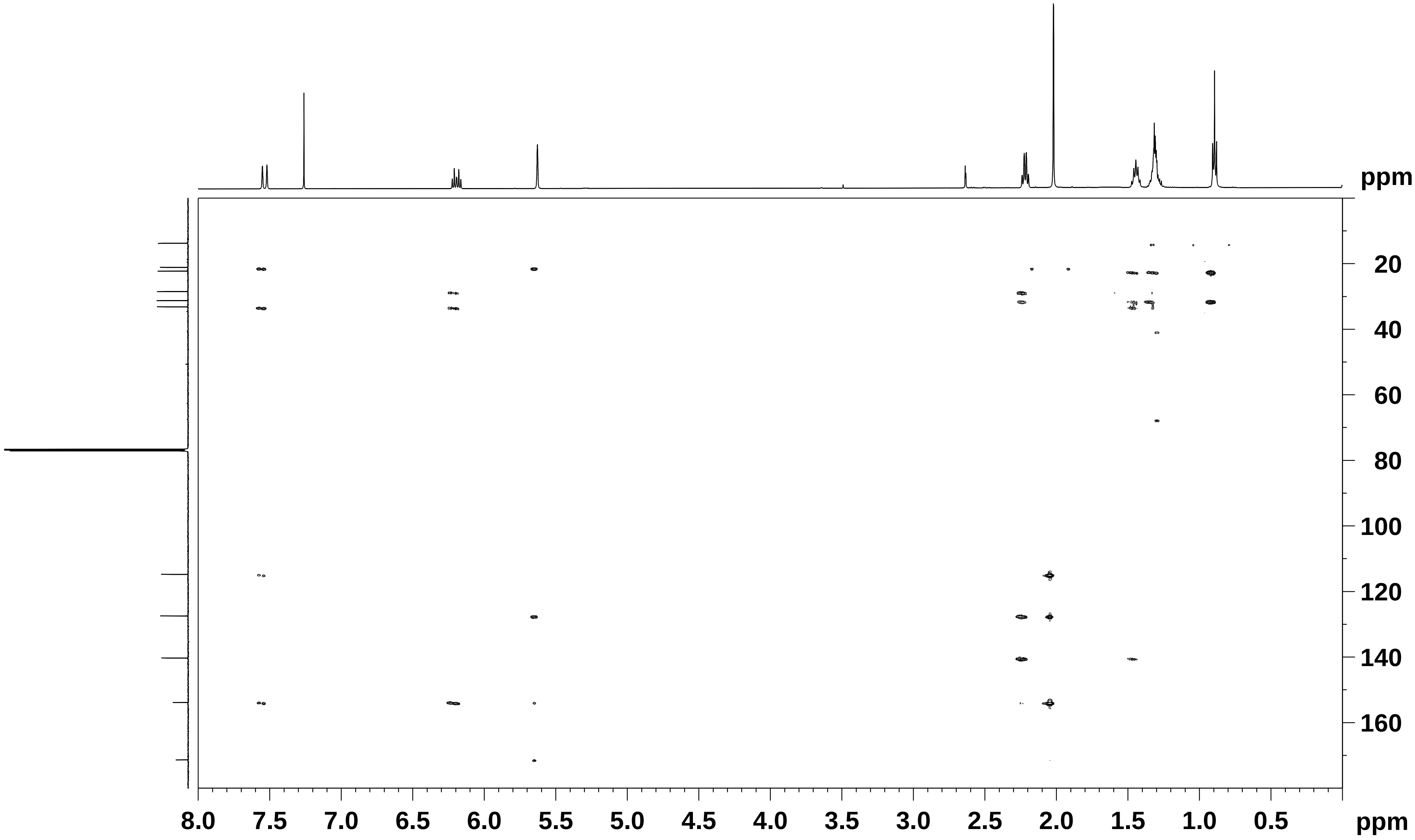

Figure S9. HMBC spectrum of **1** (500 MHz, $CDCl_3$)

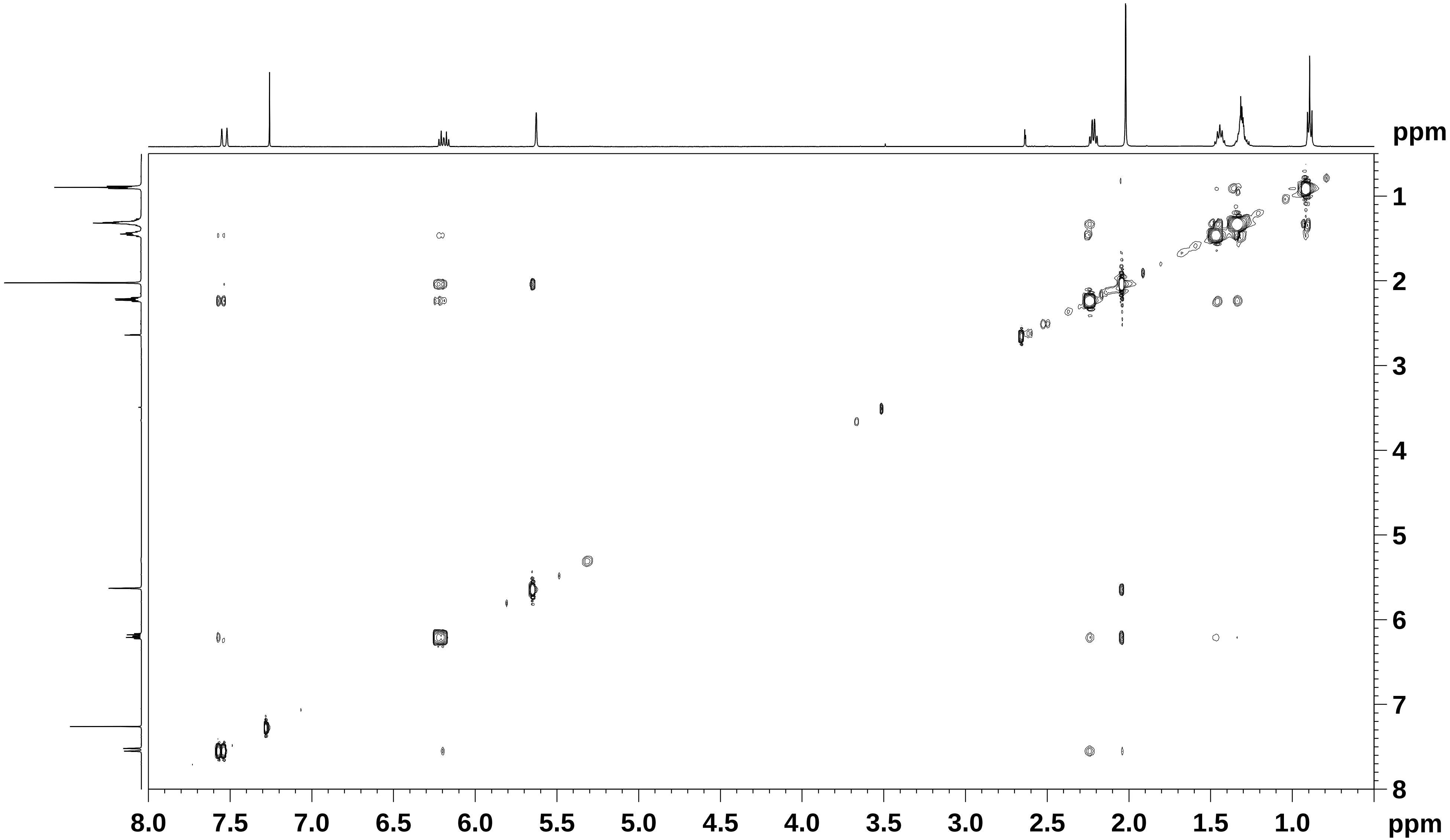

Figure S10. NOESY spectrum of **1** (500 MHz, $CDCl_3$)

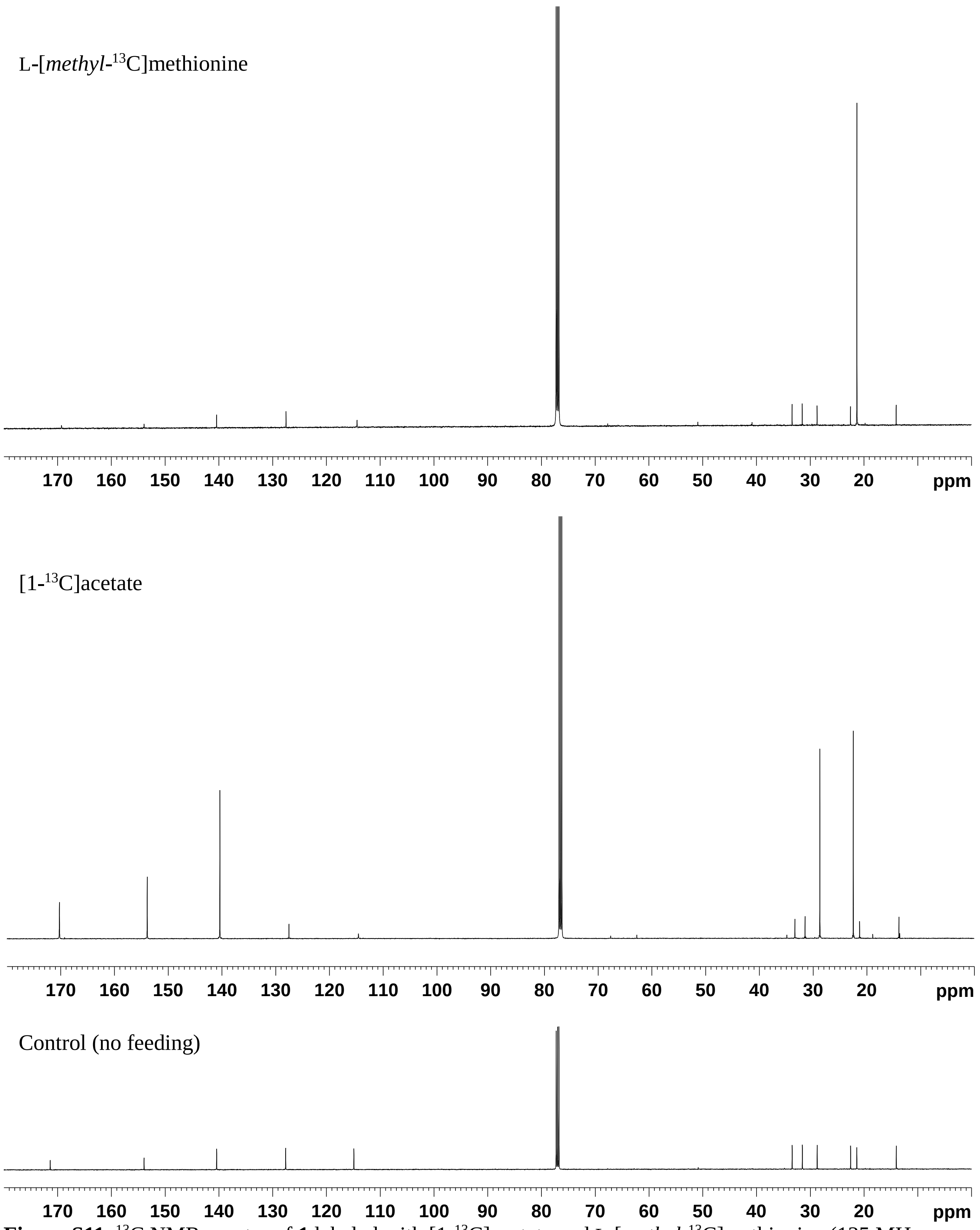

Figure S11. ^{13}C NMR spectra of **1** labeled with [1-^{13}C]acetate and L-[*methyl*-^{13}C]methionine (125 MHz, $CDCl_3$)
